# Trend change in delayed first antenatal care visit among reproductive-aged women in Ethiopia: multivariate decomposition analysis

**DOI:** 10.1186/s12978-022-01373-2

**Published:** 2022-03-28

**Authors:** Asaye Alamneh, Achenef Asmamaw, Mehari Woldemariam, Chalachew Yenew, Getaneh Atikilt, Minwuyelet Andualem, Amare Mebrat

**Affiliations:** 1 Present Address: Department of Social and Public Health, College of Health Science, Debre Tabor University, Debre Tabor, Ethiopia; 2 Department of Epidemiology and Biostatistics, Institute of Public Health, College of Medicine and Health Science, University of Gondar, Gondar, Ethiopia; 3 Department of English Language and Literature, Faculty of Social Science and Humanities, Debre Tabor University, Debre Tabor, Ethiopia; 4 Department Epidemiology and Biostatistics, Institute of Public Health, College of Health Science, Woldia University, Woldia, Ethiopia

**Keywords:** Decomposition analysis, Delayed antenatal, Ethiopia, Trends

## Abstract

**Background:**

Early first antenatal care visit is a critical health care service for the well-being of women and newborn babies. However, many women in Ethiopia still start their first antenatal care visit late. We aimed to examine the trend in delayed first antenatal care visit and identify the contributing factors for the trend change in delayed first antenatal care visits in Ethiopia over the study period 2000–2016.

**Method:**

We analyzed the data on reproductive-aged women from the four consecutive Ethiopian Demographic and Health Surveys to determine the magnitude and trend of delayed first antenatal care visit. A weighted sample of 2146 in 2000, 2051 in 2005, 3368 in 2011, and 4740 women in 2016 EDHS were involved in this study. All statistical analysis was undertaken using STATA 14. Multivariate logistic decomposition analysis was used to analyze the trends of delayed first antenatal care visit over time and the contributing factors to the change in delayed first antenatal care visit.

**Results:**

The prevalence of delayed first antenatal care visit in Ethiopia decreased significantly from 76.8% (95% CI 75.1−78.6) in 2000 to 67.3% (95% CI 65.9−68.6) in 2016. Decomposition analysis revealed that 39% of the overall change in delayed first antenatal care visit overtime was due to differences in women’s composition, whereas 61% was due to women’s behavioral changes. In this study, residence, husband's education, maternal occupation, ever told about pregnancy complications, cesarean delivery and family sizes were significantly contributing factors for the decline in delayed first antenatal care visit over the study periods.

**Conclusion:**

The prevalence of delayed first antenatal care visit in Ethiopia among women decreased significantly over time. More than halves (61%) decline in delayed first antenatal care visits was due to women’s behavioral changes. Public health interventions targeting rural residents, poor household economic status and improving awareness about pregnancy-related complications would help to reduce the prevalence of delayed first antenatal care visit.

## Plain Language summary

Antenatal care visits during the first trimester of the pregnancy stage provide opportunities for women to improve their health, prevent disease, and identify and manage pregnancy and childbirth complications. However, delayed first antenatal care visit is a major leading cause of pregnancy and childbirth complications, and many women in Ethiopia still start ANC visits late. Hence, we aimed to examine the trends in delayed first antenatal care visits and identify the contributing factors for the trend change in delayed first antenatal care visits in Ethiopia over the study period (2000–2016). We obtained data on women aged 15–49 years from four consecutive Ethiopian Demographic and Health Surveys (EDHSs) conducted in 2000, 2005, 2011, and 2016. We extracted both response and independent variables for this study from the Kids Record (KR file) data set. The prevalence of delayed first ANC visit has significantly decreased from 76.8% [95% CI 75.1, 78.6] in 2000 to 67.3% [95% CI 65.9, 68.6] in 2016 over the last sixteen years in Ethiopia. The overall trend change in delayed first antenatal care visits over the study periods was due to the difference in women's composition of selected characteristics and women's behavioral changes on ANC initiation. More than halves (61%) decline in delayed first ANC visits among reproductive-aged women was due to women's behavioral change, and 39% was due to change in women's composition over time. The significant contributing factors for the decline in delayed first antenatal care visits over the study period were residence, husband's educational level, mass media exposure, ever told about pregnancy complications, cesarean delivery and family sizes. Changes in the composition of women's characteristics according to residence areas, media exposure, ever told about pregnancy complications, cesarean delivery and family sizes were statistically significant variables for the decline in delayed first ANC visit over time. Only husbands who had secondary and above education were attributable to women’s behavioral change on ANC services.

## Background

Maternal health is the well-being of women before pregnancy, during pregnancy, childbirth and the postpartum period. Globally, the maternal mortality ratio was decreased by 44% between 1990 and 2015, in which 810 women die every day [1], and approximately 94% of maternal death occurred from preventable causes of pregnancy and childbirth complications in developing countries, including Ethiopia [2, 3]. Based on the Ethiopian Demographic and Health Survey (EDHS) reports, the maternal pregnancy-related mortality ratio decreased from 871 per 100,000 in 2000 to 412 per 100,000 live births in 2016, which was sluggish progress [4]. Although the Sustainable Development Goals (SDGs) agenda pledged to reduce the maternal mortality ratio (MMR) to less than 70 per 100,000 live births between 2016 and 2030 [5]; however, it is still cumbersome to achieve the ultimate goal in this agenda issue. Therefore, timely or early initiation of maternal health services will improve pregnancy outcomes and reduce pregnancy-related mortality rates through early treating and preventing the underlying causes of pregnancy and childbirth complications.

Many maternal health problems are preventable and treatable if they access essential health care services, for instance, ANC adequately. Antenatal care (ANC) is an entry point for women in the health care system that allows sufficient time for essential interventions, early identification of pregnancy complications, and prevention of the underlying causes of maternal and newborn mortality [6, 7]. The World Health Organization (WHO) altered the minimum number of focused ANC visits from four to eight contacts, with the first ANC visit should be taken place within the first 12 weeks of gestational age [3, 6]. In Ethiopia, the Federal Ministry of Health (FMOH) recommends the first ANC visit to be commenced within 12 weeks or four months of pregnancy [8].

First-trimester of the pregnancy stage is the fastest fetus developmental period, in which all parts of the body will be well developed and need special attention [9]. Early ANC visit allows health care providers to counsel women to deliver in the health facility [6] and to treat pregnancy-related complications like gestational diabetes, sexually transmitted infections (human immunodeficiency virus, or syphilis), and anemia for better maternal and neonatal health [10, 11]. Early booking of ANC also provides a better hemoglobin concentration through timely iron foliate acid supplementation, nutritional advice, and early treatment of malaria [11, 12].

To improve access to essential health care services, all government health institutions in Ethiopia provide ANC services free of charge for all pregnant women. Health extension workers and health professionals provide health care services in both urban and rural areas to improve the health of mothers and newborn babies through early initiation of ANC visits and mobilizing the community to utilize ANC services [12, 13]. In Ethiopia, 62% of pregnant women attended ANC visits at least once, and 26% of these women started their first ANC visit during the four to the fifth months [4].

Despite the improvement achieved in maternal health services accessibility, the time women start their first ANC visit is a well-known challenge to provide frequent and adequate ANC service in Ethiopia. Still, many women attain their first ANC visit with the pregnancy already compromised or at more risk of acquiring genitourinary tract infections, HIV, malaria, syphilis, anemia, and hepatitis B virus [4, 14–16].

The Anderson-Newman behavioral model for health services utilization provides a framework to identify factors that influence the individuals to use available health care services and classify identified factors into three parts such as predisposing, enabling, and needing variables [17, 18]. Previous studies conducted in different countries have identified various factors like; rural residence, maternal education, parity, ever told about pregnancy complications, listening to radio and television, and maternal obstetric factors associated with delayed first ANC visit [11, 19–24].

However, no evidence showed studies conducted at a national level to examine the trends and identify the contributing factors for the trend change in delayed first ANC visit via decomposition analysis. The finding will help policymakers implement an intervention on the contributing factors to the trend change in delayed first ANC visit prevalence. Moreover, it also gives direction for concerned bodies to focus on different strategies to promote maternal and neonatal health. Therefore, the objective of this study is to examine the trend and identify the contributing factors for a trend change in delayed first ANC visit over time using multivariate decomposition analysis based on 2000–2016 EDHSs.

## Method and materials

### Data source and population

This study entailed 2000, 2005, 2011, and 2016 Ethiopian Demographic and Health Surveys (EDHSs) data on reproductive-aged women, and these EDHSs were nationally representative surveys conducted in nine regions and two administrative cities. The survey used a two-stage stratified cluster sampling design to select respondents by separating each region into rural and urban areas; a total of 21 sampling designs or strata were created.

In the first stage, a total of 539 clusters (401 in rural areas and 138 in urban areas) for EDHS 2000, 540 clusters (395 in rural areas and 145 in urban areas) for EDHS 2005, 624 clusters (437 in rural areas and 187 in urban areas) for EDHS 2011 and 645 clusters (443 in rural areas and 220 in urban areas) for EDHS 2016 were selected with proportional allocation to cluster size.

**Fig. 1:**
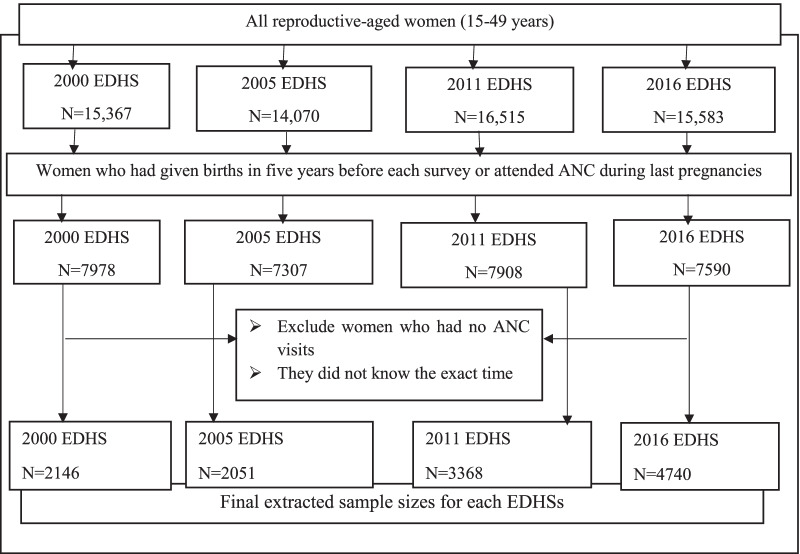
The extracted sample sizes from four consecutive EDHSs

In the second stage, household listing operations were performed in all selected clusters. On average, 27 to 32 households per cluster were selected proportional to the cluster size. We extracted relevant factors for this study from the Kids Record (KR file) dataset. A total of weighted sample 2146 in 2000, 2051 in 2005, 3368 in 2011, and 4740 in 2016 under reproductive-aged women were included in the study (Fig. 1). Comprehensive sampling procedures were described in the EDHS report [4, 25–27].

### Study population

All reproductive-aged women who had been given births or attended antenatal care during the last pregnancy within five years before the survey in all selected clusters in Ethiopia were the study population. Nonetheless, women who did not know the exact time of their first ANC visit were excluded from this study.

### Study variables

#### Outcome variable

Women asked to report the exact time of their first antenatal care visit (in months) in each survey. Our outcome variable was a delayed first ANC visit, which was determined based on the timing of the first ANC visit [4, 8].

The binary response variable for women is denoted by a random variable Yi, with two possible codes. The two possible values coded as Y_i_ = 1 if ith women started their first ANC visit after four months and Y_i_ = 0 if they started their first ANC visit before four months or at four months.

### Independent variables

We classified the independent variables in the study based on Andersen-Newman's behavioral model for maternal health care utilization as predisposing, enabling, and need factors. In the first category, predisposing variables are socio-demographic and socio-cultural characteristics of the respondents that exist before their health condition. Some predisposing factors are maternal age, marital status, residence, religion, women's occupational status, husband's occupational status, women's educational level, husband's educational level, mass media exposure, parity, family sizes and living children in a household.

In the second category, enabling (economic) factors reflect the means or facilitators to access health care services like household head and wealth index. In the last section, needing factors are the immediate causes to use health services and reflect the perceived health status of the respondents. Ever use contraceptive in a previous pregnancy, cesarean delivery of last births, wanted last pregnancy, told about pregnancy complications last births or a previous pregnancy, and ever had a terminated pregnancy (miscarriage, abortion, or stillbirths) are considered as needing factors.

### Data management and analysis

We extracted data on reproductive-aged women from the Kids Recode (KR file) data set. Before doing any statistical analysis, the data on women were weighted using sampling weights or pweights for probability sampling and non-response rate to restore the representativeness of the survey and get reliable statistical estimates.

Data analysis in this study included descriptive and multivariate decomposition analysis of the change in delayed first ANC visit. After extracting relevant variables for the study, we appended data on reproductive-aged women obtained from the four 2000, 2005, 2011, and 2016 EDHSs together for trends and multivariate decomposition analysis. Besides, multicollinearity was checked using variance inflation factor (VIF) and a VIF less than 5 for each independent variable. Therefore, there was no multicollinearity between independent variables since its VIF value ranged from 1.01 to 2.15 with a mean VIF of 1.4.

### Trends and decomposition analysis

The trend period was divided into four phases such as; first phase (2000–2005), second phase (2005–2011), third phase (2011–2016), and fourth phase (2000–2016) to see the differences in the prevalence of delayed first ANC visit over time-based on different selected characteristics of women. The trend was assessed using descriptive analysis stratified by various selected predictor variables and examined separately for each phase.

Multivariate decomposition analysis was used to identify the contributing factors to the trend change in the outcome variable between any two surveys over time. This analysis focused on how a delayed first ANC visit prevalence responds to differences in selected women's characteristics and how these variables shape the differences across the surveys conducted at different times. Decomposition analysis aimed to identify the potential sources of variations in the prevalence of delayed first ANC visit in the last 16 years. Multivariate decomposition analysis for the non-linear response model used the output from a logistic regression model since it is a dichotomous variable to parcel out the observed change in delayed first ANC visit between surveys into components. The difference in the percentage of delayed first ANC visit over time is attributable to the compositional change between any two surveys and the difference in the effects of those selected independent variables. That means the change in delayed first ANC visit is divided into the differences in characteristics (endowment component) and the effect of the selected variables (coefficient component). The analysis focused on the decomposition of the trend change in delayed first ANC visit between the reference year (2000) and the recent year (2016). The recent EDHS 2016 and reference EDHS 2000 surveys are denoted by A and B, respectively.

For logistic regression, log-odds or logit of delayed first ANC visit divided into two main parts as follow: 
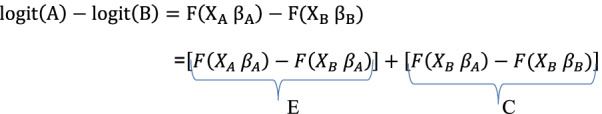
where E represents endowments explained by characteristics, and C denotes coefficients (unexplained) [28].

We can rewrite the above equation as follow:

$${\text{logit}}\left( A \right) - {\text{logit}}\left( B \right) = \left[ {\beta_{0A} - \beta_{0B} } \right] + \sum X_{ijB} *\left[ {\beta_{ijA} - \beta_{ijB} } \right] + \sum {\upbeta }_{ijB} *\left[ {X_{ijA} - X_{ijB} } \right]$$

where $\beta_{0B}$ is the intercept in the regression equation for EDHS 2000,

$\beta_{0A}$ is the intercept in the regression equation for EDHS 2016,

$\beta_{ijB}$ is the coefficient of the $jth$ category of the $ith$ determinant in EDHS 2000,

$\beta_{ijA }$ is the coefficient of the $jth$ category of the $ith$ determinant in EDHS 2016,

$X_{ijB}$ is the proportion of the $jth$ category of the $ith$ determinant in EDHS 2000, and

$X_{ijA}$ is the proportion of the $jth$ category of the $ith$ determinant in EDHS 2016.

Currently developed multivariate logistic decomposition analysis used for the decomposition analysis of delayed first ANC visit using mvdcmp STATA package [29].

## Results

### Characteristics of the study population

Based on maternal age, women aged 29–30 years constituted approximately 67% of women from the dataset. More than four-fifths (80%) in all surveys were male household heads (Table 1).

**Table 1:** Characteristics and percentage distribution of women in Ethiopia

Characteristics and their categories	Percentage distribution of the surveys			
	EDHS 2000	EDHS 2005	EDHS 2011	EDHS 2016
	N = 2146	N = 2051	N = 3368	N = 4740
Maternal age				
< 20 years	6.6	5.7	4.7	5.3
20–34 years	69.0	71.6	71.9	73.1
≥ 35 years	24.4	22.7	23.4	21.6
Household head				
Female	17.6	14.9	18.2	14.6
Residence				
Rural	71.7	78.5	73.1	81.6
Women educational level				
No education	64.5	62.3	52.9	53.9
Primary education	21.2	23.1	37.2	33.2
Secondary & above	14.3	14.6	9.9	12.9
Husband educational level				
No education	48.0	41.5	37.2	40.1
Primary education	28.4	33.2	45.9	40.8
Secondary & above	23.6	25.3	16.9	19.1
Maternal occupation status				
Not working	34.1	64.3	41.5	50.7
Husband occupation status				
Not working	0.5	0.9	0.9	5.8
Reading newspapers				
No	84.0	85.3	83.2	90.0
Listening to a radio				
No	56.8	49.2	37.3	33.8
Watching television				
No	84.6	78.9	52.4	74.7
Ever told about pregnancy complications				
No	72.7	68.3	79.7	55.0
Ever had terminated pregnancy				
No	83.6	89.9	89.2	90.8
Wantedness of pregnancy				
Wanted then	56.9	60.3	68.0	76.0
Wanted later	21.3	21.5	21.7	17.3
Wanted no more	21.8	18.2	10.3	6.7
Cesarean delivery				
No	97.8	96.3	96.0	96.5
Parity				
1	37.7	40.0	41.1	41.2
2–4	37.7	34.3	36.8	36.4
≥ 5	24.6	25.7	22.1	22.4
Family size				
≤ 2	2.5	1.9	3.3	2.1
3–4	28.3	30.4	29.3	33.1
≥ 5	69.2	67.7	67.4	64.8
Ever use contraceptive				
No	81.6	75.3	61.6	58.1
Region				
Tigray	9.1	8.7	10.1	10.1
Afar	1.1	0.6	0.8	0.8
Amhara	19.3	23.8	23.7	23.0
Oromia	39.5	33.4	36.4	33.8
Somali	0.6	1.1	1.5	2.5
Benshangul-Gumuz	1.0	0.8	1.1	1.2
SNNP	22.1	24.6	9.7	23.4
Gambla	0.6	0.4	0.5	0.3
Harari	0.4	0.3	0.3	0.3
Addis Ababa	5.6	0.5	5.4	4.0
Dire Dawa	0.7	0.6	0.5	0.6

Regarding maternal educational level, the proportion of women who had no formal education showed a high decline from 64.5% in 2000 to 53.9% in 2016. However, the proportion of women who had primary education was 21.2% in 2000 and 33.2% in 2016. But about 48% and 40.1% of their husbands in 2000 and 2016 had no formal education, respectively. Based on the place of residence, approximately 75% of the women sampled from all surveys were rural residents. In four consecutive surveys, most women (> 80%) had no previous terminated pregnancy over time.

Across the four EDHS surveys, the proportion of women who wanted last pregnancy then (that time) increased from 56.9% in 2000 to 76% in 2016. However, the proportion of women who wanted last pregnancy later declined from 21.3% in 2000 to 17.3% in 2016, while women who wanted no more last pregnancy decreased from 21.8% to 6.7%. Additionally, the proportion of women told about pregnancy-related complications rose from 27.3% in 2000 to 45.0% in 2016 over the two study periods (Table 1).

### Trends in delayed first ANC visit

**Fig. 2:**
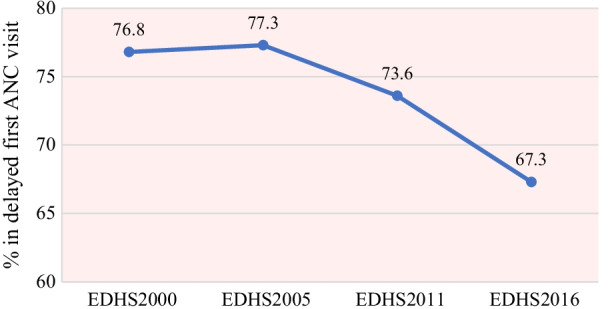
The trends of delayed first ANC visit in Ethiopia from 2000 to 2016

The prevalence of delayed first ANC visit among reproductive-aged women in Ethiopia decreased from 76.8% in 2000 to 67.3% in 2016 in the last 16 years. The trends of delayed first ANC visit over the study periods (2000–2016) were divided into four phases such as 2000–2005, 2005–2011, 2011–2016 and 2000–2016 to see the differences in delayed first ANC visit over time and the potential sources for the change in delayed first ANC visit. The highest decrement was observed in the fourth phase (2000–2016) with a 9.5% point change in delayed first ANC visit compared with 3.7% and 6.7% point change in delayed first ANC visit in the second phase and third phase, respectively. The prevalence of delayed first ANC visit declined from 76.8% [95% CI 75.1, 78.6] in 2000 to 67.3% [95% CI 65.9, 68.6] in 2016 (p-value < 0.001), with a 9.5% overall point change (Fig. 2).

### Trends in the prevalence of delayed first ANC visit by selected variables

Trends of delayed first ANC visit among reproductive-aged women showed variation based on different characteristics. Delayed first ANC visit prevalence decreased in most of the categories of the variables in each phase (Table 2).

**Table 2:** Trends in the prevalence of delayed first ANC visits among women by selected characteristics between 2000 and 2016 Ethiopia Demographic and Health Surveys

Characteristics	EDHS 2000	EDHS 2005	EDHS 2011	EDHS 2016	Percentage point difference in delayed first ANC visit			
	N = 2146	N = 2051	N = 3368	N = 4740	2000–2005	2005–2011	2011–2016	2000–2016
Delayed first ANC booking	76.8	77.3	73.6	67.3	0.5	− 3.7	− 6.3	− 9.5
Mother’s age								
< 20 years	83.4	79.7	75.4	63.5	− 3.7	− 4.3	− 12.0	− 19.9
20–34 years	76.3	75.7	73.3	67.0	− 0.7	− 2.3	− 6.3	− 9.3
≥ 35 years	76.5	81.0	74.2	69.2	4.4	− 6.8	− 5.0	− 7.34
Residence								
Rural	81.6	83.8	78.9	71.0	2.2	− 4.9	− 7.9	− 10.6
Urban	64.8	53.4	59.4	51.1	− 11.4	6.0	− 8.3	− 13.7
Household head								
Female	75.2	63.2	66.9	61.1	− 12.0	3.7	− 5.9	− 14.2
Male	77.2	79.7	75.1	68.4	2.5	− 4.6	− 6.7	− 8.8
Women education								
No education	81.4	82.42	78.4	71.2	1.0	− 4.0	− 7.2	− 10.3
Primary education	76.5	76.6	71.7	66.3	0.1	− 4.9	− 5.3	− 10.1
Secondary education	56.6	56.3	55.5	53.7	− 0.3	− 0.8	− 1.8	− 2.9
Husband education								
No education	81.7	81.84	78.4	69.5	0.1	− 3.5	− 8.9	− 12.3
Primary education	79.4	84.2	75.6	70.4	4.8	− 8.6	− 5.2	− 9.0
Secondary education	64.2	61.1	59.5	59.6	-3.1	− 1.7	0.1	− 4.6
Wanted the last pregnancy								
Wanted then	73.6	77.4	74.4	66.9	3.8	− 2.9	− 7.6	− 6.7
Wanted later	74.1	77.8	70.6	66.4	3.6	− 7.2	− 4.2	− 7.7
Wanted no more	79.1	76.1	74.5	74.5	− 3.0	− 1.6	0.1	− 4.5
Women occupation								
Working	77.5	75.4	73.6	64.3	− 2.1	− 1.8	− 9.3	− 13.3
Not working	75.5	78.3	73.7	70.2	2.8	− 4.6	− 3.5	− 5.2
Husband occupation								
Working	76.7	77.7	74.0	67.0	1.0	− 3.7	− 7.0	− 9.7
Not working	80.9	81.1	45.4	67.3	0.2	− 35.7	21.8	− 13.6
Reading newspapers								
Yes	63.8	61.1	65.3	51.7	− 2.7	4.2	− 13.6	− 12.1
No	79.3	80.1	75.3	69.0	0.8	− 4.8	− 6.3	− 10.3
Listening to radio								
Yes	71.0	70.4	72.5	65.2	− 0.6	2.1	− 7.3	− 5.8
No	81.3	84.3	75.6	68.4	3.1	− 8.8	− 7.2	− 12.9
Watching television								
Yes	60.9	55.8	68.8	54.8	-5.1	13.0	− 14.0	− 6.1
No	79.8	83.0	78.0	71.5	3.2	− 5.0	− 6.5	− 8.3
Told pregnancy complication								
Yes	73.5	67.2	64.1	63.3	− 6.3	− 3.1	− 0.8	− 10.2
No	78.2	81.8	76.2	70.7	3.6	− 5.7	− 5.6	− 7.6
Ever terminated pregnancy								
Yes	75.9	69.5	80.0	69.2	− 9.4	10.5	− 10.8	− 6.7
No	77.0	78.1	72.8	67.1	1.1	− 5.3	− 5.7	− 9.9
Cesarean delivery								
Yes	57.6	49.0	63.8	37.2	− 8.6	14.8	− 26.6	− 20.4
No	77.3	78.3	74.0	68.4	1.0	− 4.3	− 5.6	− 8.9
Parity								
1	76.6	72.0	69.4	62.8	− 4.6	− 2.6	− 6.6	− 13.8
2–4	77.4	79.1	76.1	66.9	1.7	− 3.0	− 9.2	− 10.5
≥ 5	76.3	82.9	77.2	76.2	6.6	− 5.7	− 1.0	− 0.1
Family size								
≤ 2	65.0	58.8	69.3	32.4	− 6.1	10.5	− 36.8	− 32.5
3–4	77.1	73.5	69.0	64.3	− 3.4	− 4.6	− 4.7	− 12.8
≥ 5	77.1	79.4	75.9	69.9	2.3	− 3.6	− 5.9	− 7.2
Ever use contraceptive								
Yes	62.2	67.8	68.1	63.3	5.6	0.3	− 4.8	1.1
No	80.1	80.4	77.1	70.2	0.2	− 3.3	− 6.9	− 10.0
Region								
Tigray	82.2	80.0	75.8	63.0	− 2.2	− 4.2	− 12.8	− 19.2
Afar	80.6	73.6	68.9	63.2	− 7.0	− 4.7	− 5.7	− 17.4
Amhara	77.5	77.6	73.9	58.5	0.1	− 3.7	− 15.5	− 19.0
Oromia	74.6	75.5	75.9	71.2	0.9	0.4	− 4.7	− 3.4
Somalia	69.8	49.7	63.6	68.6	− 20.1	13.9	5.0	− 1.1
Benshangul-Gumuz	81.8	82.2	84.3	76.9	0.4	2.2	− 7.4	− 4.8
SNNPR	84.3	87.0	77.3	78.3	2.6	− 9.7	1	− 6.1
Gambela	67.3	73.1	57.7	55.9	5.8	− 15.4	− 1.7	− 11.4
Harari	59.7	55.7	44.6	41.4	− 3.9	− 11.1	− 3.2	− 18.2
Addis Ababa	56.5	49.1	47.0	37.6	− 7.4	− 2.0	− 9.4	− 18.9
Dire Dawa	56.0	46.1	42.0	31.1	− 9.9	− 4.1	− 10.9	− 24.9

The prevalence of delayed first ANC visit decreased by 10.6% among rural residents during the fourth phase (2000–2016). Based on region, delayed first ANC visit prevalence decreased in the last period (2000–2016) at a 19.0% point change in delayed first ANC visit (Table 2). The highest difference in delayed first ANC visit was in Dire-Dawa (24.9%), but the least 1.1% point change in delayed first ANC visit was in Somali (Table 2).

According to maternal educational status, there was a decrease in the prevalence of delayed first ANC visit among women with primary education in the last phase (2000–2016) by a 10.1% point change. Additionally, women who had only two family members and family sizes between three and four in a household showed a decrement in delayed first ANC visit prevalence with 32.5% and 12.8% point change, respectively (Table 2).

### Decomposition analysis

#### Decomposition analysis of delayed first ANC visit in Ethiopia, 2000–2016

Overall, there has been a significant decrease in delayed first ANC visit prevalence among reproductive-aged women in Ethiopia. The decomposition results showed that the decline in delayed first ANC visit over time was explained by the difference in the selected women’s characteristics and behavioral changes between the two survey points. 39% of the decrease in delayed first ANC visit prevalence was due to the differences in the composition of characteristics; the change was due to the differences in the effect of the selected variables was 61% (Table 3).

**Table 3:** Overall decomposition of the change in delayed first ANC visit among reproductive-aged women in Ethiopia 2000–2016

Components	Coeff (95% CI)	Pct
Difference in characteristics (E)	− 0.032592 (− 0.056518, − 0.008665)	39.1
Difference in coefficients (C)	− 0.05091 (− 0.089767, − 0.012034)	60.9
Residual (R)	− 0.083492 (− 0.11491, − 0.052073)	

### Difference due to characteristics (endowment)

Multivariate decomposition analysis result revealed that the overall decrease in delayed first ANC visit was due to the difference in characteristics (the differences in the composition of women's selected variables) between the two survey points (Table 3). In this study: residence, listening to the radio, watching television, ever told about pregnancy complications, cesarean delivery, and family members in the household showed a significant effect for the decline in delayed first ANC visit (Table 4).

**Table 4:** Detailed decomposition of the change in delayed first ANC visit among reproductive-aged women in Ethiopia 2000–2016

Variables	Difference due to characteristics (E)		Difference due to coefficients (C)	
	Coeff (95% CI)	Pct	Coeff (95%CI)	Pct
Household head				
Female	0.0002138 (− 0.002953, 0.003380)	− 0.3	− 0.04131 (− 0.018035, 0.009773)	4.9
Residence				
Rural	0.006818 ^b^ (0.00095109, 0.012685)	− 8.2	0.0006295 (− 0.048803, 0.0747)	− 15.5
Reading newspapers				
No	− 0.0036942 (− 0.008142, 0.0007534)	4.4	− 0.029002 (− 0.18088, 0.12287)	34.7
Listening to a radio				
No	− 0.04351^b^ (− 0.008333, − 0.0003689)	5.2	0.018395 (− 0.012632, 0.049422)	− 22.0
Watching to television				
No	− 0.007204^b^ (− 0.012087, − 0.0023201)	8.6	− 0.005452 (− 0.019421, 0.008516)	6.5
Ever terminated pregnancy				
No	− 0.0003671 (− 0.004347, 0.003613)	0.4	0.002952 (− 0.010704, 0.016608)	− 3.5
Told about pregnancy complications				
No	− 0.0066515^b^ (− 0.012924, − 0.0003792)	8.0	0.030205 (− 0.015242, 0.075651)	− 36.2
Cesarean delivery				
No	− 0.002201^b^ (− 0.000355, − 0.0000852)	0.3	− 0.002895 (− 0.007066, 0.001276)	3.5
Wantedness of pregnancy				
Wanted then^a^				
Wanted later	− 0.0000595 (− 0.001919, 0.001800)	0.1	0.0061642 (− 0.010147, 0.022476)	− 7.4
Want no more	− 0.007346 (− 0.01921, 0.0045191)	8.8	0.014025 (− 0.0064513, 0.034501)	− 16.8
Women education level				
No education^a^				
Primary education	− 0.001951 (− 0.007301, 0.003407)	2.3	0.0007179 (− 0.015125, 0.01656)	− 0.9
Secondary and above education	0.000176 (− 0.000494, 0.000845	− 0.2	0.013158 (− 0.0024387, 0.028755)	− 15.8
Husband education level				
No education^a^				
Primary education	0.003822 (− 0.0014566, 0.0091)	− 4.6	0.009099 (− 0.011995, 0.030193)	− 10.9
Secondary education & above	− 0.002262 (− 0.004877, 0.000354)	2.7	0.022655^b^ (0.0001921, 0.04512)	− 27.1
Parity				
1^a^				
2–4	5.792e−05 (− 0.0005397, 0.0006556)	− 0.1	0.002656 (− 0.034132, 0.039443)	− 3.2
≥ 5	− 0.0007894 (− 0.001665, 0.00008664)	0.9	0.033711 (− 0.002834, 0.070257)	− 40.4
Family size				
≤ 2^a^				
3–4	0.0096351^b^ (0.0027093, 0.016561)	− 11.5	0.024385 (− 0.035153, 0.083923)	− 29.2
≥ 5	− 0.056901^b^ (− 0.010004, − 0.001376)	6.8	0.047305 (− 0.098709, 0.19332)	− 56.7
Ever contraceptive use				
No	− 0.008472 (− 0.017782, 0.000837)	10.2	0.008745 (− 0.004505, 0.02199)	− 10.5
Constant			− 0.24466 (− 0.48512, − 0.0041945)	293

^a^Reference; ^b^significant at 5% level of significance; Pct, percent; Coef, coefficient

From predisposing factors, multivariate decomposition analysis showed that residence, listening to the radio, and family members were statistically significant variables for the change in delayed first ANC visit. The increment in the proportion of rural residents in the sample showed a significant 8.3% contribution to the decline in delayed first ANC visit. A decrease in the composition of women who had three up to four-family sizes in the sample showed a significant negative impact on the change in delayed first ANC visit by 11.5%; another 6.8% was due to a decrease in the composition of women who had five and above family sizes in the household (Table 4). A decrease in the proportion of women with no exposure to listening to the radio over time (from 2000 to 2016) significantly contributed to the decline in delay first ANC visit, which contributes 5.2% (Table 4).

Of all enabling factors: ever told about pregnancy complications and cesarean delivery were significant factors to a decline in delayed first ANC visit. The compositional change in women with ever told about pregnancy complications in the sample showed a significant contribution to the decrease in the prevalence of delayed the first ANC visit by 8%. Women who did not experience cesarean delivery in the sample showed a significant positive 0.3% contribution to the decrease in delayed first ANC visit (Table 4).

Notable, negative signs on the percentage changes indicate that those compositional changes have a negative or reversal effect for delayed first ANC visit.

### Difference due to effects of the coefficient (Effects of characteristics)

Keeping the compositional changes constant, about 61% of the overall decline in delayed first ANC visit was due to the differences in the effect of the independent variables (coefficients) (Table 3). Of all factors included in the analysis, only the educational levels of husbands showed a significant contribution effect for the behavioral change in delayed first ANC visit. Husbands who had secondary or higher education showed a significant contribution to the observed percentage decline in delayed first ANC visit over time, which contributed 27.3% (Table 4).

## Discussion

Delayed antenatal care is a major leading cause of pregnancy-related complications and maternal and fetal health problems [2, 30, 31]. This study examined the trends and the contributing factors for the change in delayed first ANC visit in Ethiopia using the EDHS 2000–2016. The trend of delayed first ANC visit prevalence has been significantly declined from 76.8% in 2000 to 67.3% in 2016. This finding is in line with the study done in Sub-Saharan Africa and Nigeria, in which the trend estimates of delayed first ANC visit decreased over time [32, 33]. The possible reason may be the health facilities in Ethiopia provide ANC services for women free of charge; health care providers create awareness for the community about the benefits of early ANC booking for the well-being of women and fetuses [12]. Moreover, Health Development Armies (HDAs) are launching the Health Extension Program [33], improving access to health care to meet the primary goals of the MDGs agenda, and introducing an integrated community case management program [34].

In the decomposition analysis, the prevalence of delayed first ANC visit declined significantly over time. Therefore, understanding the potential sources of differences has public health importance to know what are the contributing factors for the change in delayed first ANC visit and to evaluate already implemented strategies. About 61% of the overall decline in delayed first ANC visit over the last sixteen years was due to behavioral changes among women about ANC booking. However, 39% of the decrement in delayed first ANC visit was due to the differences in women's composition over time.

An increment in the proportion of rural resident women from 2000 to 2016 revealed a significant negative impact on the decline in delayed first ANC visits and contributed about 8.2% of the changes. This finding is consistent with other studies in Zambia, and Vietnam [19, 35], which indicates women who lived in rural areas attend late ANC visit and inadequate frequency of ANC visits. The possible reason might be lack of accessibility to health care services, limited finances, unavailability of health facilities and professionals, lack of transportation, and no better exposure to health information in rural areas [36]. Besides, women who lived in rural areas were less likely to book early for their first ANC visit because of inadequate accessibility of health services and no equal distribution of health facilities and professionals between rural and urban areas.

An increment in the composition of women who were not working showed a significant negative impact on the change in delayed first ANC visit. This finding is consistent with a previous study in Addis Ababa, Ethiopia, in which women who had no job were more likely to book late for their first ANC visit [37]. The possible reason might be women who had no work are economically dependent and unable to cover the payment of maternal health services recommended by health workers [38]. In addition, women miss the opportunities to early receive various benefits of early ANC visits from others in the workplace.

A decrement in the composition of women told about pregnancy complications showed a significant positive effect on the decline in delayed first ANC visit. This finding is in line with prior studies in Malaysia and South Gondar, Ethiopia [39, 40]. The possible reason could be women have already been told about pregnancy-related complications and ANC initiation time in the previous pregnancy from health professionals. Moreover, the commencement of health education in their previous pregnancy may improve ANC initiation time and provide various health promotion services [21, 41].

The composition changes of the family sizes in the household showed both negative and positive contributions to the decline in delayed first ANC visit. An increment in the proportion of women who had three and four family members in a household showed a significant negative impact on the change in delayed ANC visit. A decrement in the composition of women who had five and above family members in a household showed a significant positive effect on the decline in delayed first ANC visit. This finding is consistent with prior studies in Cameroon and Ethiopia [42, 43]. The possible reason might be women who had higher family members attain late antenatal visits due to time constraints. Furthermore, this also could be they are too busy in childcare responsibility to start early first ANC visit due to having higher family members. Besides, resource constraint is another challenge to treat and manage pregnancy and childbirth complications earlier [44]. This result indicated that having higher family members in a household would increase their confidence to give birth without booking ANC visit early.

A decreasing in the proportion of women's accessibility of media exposure had a significant impact on the change in delayed first ANC visit. This finding is consistent with other studies done in North India, South India, and Northern Ethiopia [45–47]. It implies that media accessibility might have brought increased knowledge and awareness of the various benefit of early first ANC visit.

A decreasing in the composition of cesarean section delivery over time had a significant effect on the decline in delayed first ANC visits. This finding is corporate with a previous study in Australia [48]. The reason could be cesarean delivery done in health facilities, and women might have ever been told about pregnancy-related complications and the risk of ectopic pregnancy [49].

Behavioral changes of husbands or partners who had secondary and above education showed a significant positive contribution to the decline in delayed first ANC visit. This finding is consistent with other studies in Ghana and Jamaica [50, 51] authors argued that education might have improved awareness of maternal health care services utilization in general and the importance of early ANC visit in particular. Additionally, educated husbands have better knowledge about adverse consequences of delayed first ANC visit [52]. The result indicates that education enhances the self-esteem and authority of women, their negotiation skills with husbands about quality health services.

As a limitation of this study, we did not examine some factors from each survey (for example, health insurance, distance to the health facility, and accessibility and quality of health services) to perform the analysis. Thus, this study could be used as input information in future researches, and future researchers may include other factors that influence delayed first ANC visit. Decomposition analysis is a prominent tool to analyze the contributing factors for the change in delayed first ANC visit. Our model is limited to the available data in EDHS datasets to explain the difference. We recommend future researchers include another alternative method to the decomposition analysis.

## Conclusion

Delayed first ANC visit prevalence had shown a significant decrease over the last 16 years. More than half decline in delayed first ANC visit was due to the change in respondents’ behavior about health care services. Compositional changes in women's selected characteristics like residence place, husband education, ever told about pregnancy complications, cesarean delivery, family size, and watching television and listening to the radio were significant contributing factors for the decline in delayed first ANC visit.

It suggested that the increased efficiency of the health care system within society suffers from women's characteristics. Increasing women’s awareness of pregnancy complications and symptoms and leading causes for poor pregnancy outcomes is highly recommended. Moreover, public health interventions like health education and community communication education for women could decrease delayed first ANC visits, particularly for rural residents.

## Data Availability

The data are from the Measure DHS and are online publicly available domain at (https://dhsprogram.com/data/).

## Abbreviations

ANC: Antenatal Care
CI: Confidence Interval
HDA: Health Development Army
EDHS: Ethiopian Demographic and Health Survey
HEP: Health Extension Program
MDGs: Millennium Development Goals
MMR: Maternal Mortality Ratio
USAID: U.S. Agency for International Development
VIF: Variance Inflation Factor
WDA: Women Development Army
WHO: World Health Organization
