# Correction: Trend change in delayed first antenatal care visit among reproductive‑aged women in ethiopia: multivariate decomposition analysis

**DOI:** 10.1186/s12978-025-02224-6

**Published:** 2026-01-27

**Authors:** Asaye Alamneh Gebeyehu, Achenef Asmamaw Muche, Mehari Woldemariam, Chalachew Yenew, Getaneh Atikilt, Minwuyelet Andualem, Amare Mebrat

**Affiliations:** 1 Present Address: Department of Social and Public Health, College of Health, Science, Debre Tabor University, Debre Tabor, Ethiopia; 2 Department of Epidemiology and Biostatistics, Institute of Public Health, College of Medicine and Health Science, University of Gondar, Gondar, Ethiopia; 3 Department of English Language and Literature, Faculty of Social Science and Humanities, Debre Tabor University, Debre Tabor, Ethiopia; 4 Department Epidemiology and Biostatistics, Institute of Public Health, College of Health Science, Woldia University, Woldia, Ethiopia

**Correction: Reprod Health 19, 80 (2022)**

**https://doi.org/10.1186/s12978-022-01373-2**

In this article, the first author name is corrected from ‘Asaye Alamneh’ to ‘Asaye Alamneh Gebeyehu’ and the second author name is corrected from ‘Achenef Asmamaw’ to ‘Achenef Asmamaw Muche’.

The original article has been corrected.

